# Application of a Porcine Small Intestine Submucosa Nerve Cap for Prevention of Neuromas and Associated Pain

**DOI:** 10.1089/ten.tea.2019.0273

**Published:** 2020-05-15

**Authors:** Shahryar Tork, Jennifer Faleris, Anne Engemann, Curt Deister, Erick DeVinney, Ian L. Valerio

**Affiliations:** ^1^ Department of Plastic Surgery, Wexner Medical Center, The Ohio State University, Columbus, Ohio, USA.; ^2^ Axogen, Alachua, Florida, USA.

**Keywords:** neuroma, symptomatic neuroma, neuroma prevention, nerve cap, small intestine submucosa

## Abstract

Painful neuroma formation is a common and debilitating sequela of traumatic or oncologic nerve amputations. Studies suggest that isolating transected nerve stumps within protective caps during amputation surgery or revision procedures may assist in preventing symptomatic nerve-end neuroma formation. This study evaluated the local effects of two porcine small intestine submucosa (pSIS) nerve caps of differing configurations on a terminal nerve end in an animal model. The tibial nerves of 57 Sprague Dawley rats were transected and transposed to the lateral hind leg. The nerves were treated with one of three SIS materials, including (i) a nerve cap with spiraling chambering, termed spiral nerve cap (SNC), (ii) a nerve cap with bifurcated chambers termed chambered nerve cap (CNC), or (iii) an open tube. The surgical control consisted of nerve stumps that were not treated. Overall tissue response, axonal swirling, optical density of axons, and behavioral pain response were quantified at 8 and 12 weeks postoperatively. There were no notable differences between the performance of the SNC and CNC groups. The pSIS nerve caps mitigated aberrant axonal regeneration and decreased neuroma formation and associated pain response. These findings suggest that nerve caps with internal chambers for axonal outgrowth may improve axonal alignment, therefore reducing the likelihood of symptomatic neuroma formation.

**Impact statement:**

This study provides evidence for using nerve caps with internal structure on nerve stumps after amputation surgeries to reduce or prevent symptomatic neuromas. This study showed that porcine small intestine submucosa had a favorable remodeling profile and tissue response, illustrating that this device can be used to (i) minimize soft tissue attachments around the nerves that are capped, (ii) align axonal outgrowth to guide nerve regeneration away from aberrant neuroma formation, and (iii) act as a barrier between the nerve and external stimuli ultimately remodeling into a new soft tissue layer around the nerve stump thus decreasing symptomatic neuroma formation.

## Introduction

Painful neuroma formation is a common and debilitating sequela of traumatic or oncologic nerve amputations. Neuromas result from axonal outgrowth through Schwann cell proliferation ahead of an injury site as the cells attempt to restore axonal continuity of the disrupted nerve end(s). In severe tissue damage, the regenerating axons cannot reach their target tissue and instead form a tangled bulbous mass which causes pain, likely due to pathological interactions between axons within the neuroma^1,2^ as well as traction between the nerve and scar tissue or ischemic necrosis of the nervous tissue.^1,3,4^

Neuroma pain is typically characterized by spontaneous hyperalgesia and allodynia, which may endure after complete remodeling of the wound,^5^ and this pain can be triggered by external stimuli such as the pressure exerted by a prosthetic.^1^ In fact, significant stump pain associated with neuromas has been reported in over 80% of amputees^6^ and symptomatic neuromas are a frequent cause for reoperation in this population.^7–9^ In amputees, painful neuromas can make it difficult to mold a well-fitting prosthesis socket and can prevent successful use of a prosthetic, thus limiting mobility.

The current standard treatment options after limb amputation or an unrepaired transected nerve injury incompletely address pain-inducing neuroma formation.^10^ These standard treatments include surgical methods to remove neuromas or pharmacological therapies directed at pain management. The current standard of care for surgical management of symptomatic neuromas involves either traction neurectomy, which has a high rate of neuroma recurrence,^11,12^ or burying the nerve stump into adjacent muscle or bone.^12^ While burying the nerve stump in adjacent tissue mitigates external stimulation that causes painful sensation, this procedure can greatly complicate surgery as additional dissection of otherwise healthy tissue is required to secure the nerve stump. In addition, this technique is not always anatomically possible, it exposes the patient to further risks, and often fails to sufficiently isolate the affected nerve from external stimuli.

The success rates (typically defined as postsurgical pain reduction) for this approach can be highly variable with reports of 40–81% for burial in muscle and 33–91% for burial in bone.^13,14^ This variability is linked to the fact that neuromas still form when the nerve end is buried in muscle or bone^15–17^ and nerve stumps may be dislodged from muscles during large excursion through their range of motion. Therefore, one of the main issues with these techniques is their inability to prevent neuroma formation by preventing pathophysiological axon regeneration.^12^

Recently, several techniques have been developed with the goal of allowing the nerve to regenerate in a more normal, physiologic manner to prevent neuroma. Targeted muscle reinnervation has gained considerable traction as a reliable and effective modality for neuroma prevention,^18,19^ and it is speculated that the success of this technique is based on giving the nerves a natural target as they grow into and reinnervate the denervated muscle, thus reestablishing the natural aligned outgrowth pattern inherent to the native peripheral nervous system.^18^ However, this technique is technically difficult and requires a certain level of microsurgical skill. In addition, the identification and transection of a suitable motor nerve as a recipient guide for the proximal nerve stump is not always feasible from an anatomic or etiological standpoint.

Another technique that has grown in popularity that also allows for aligned axonal outgrowth involves suturing a long nerve graft, conduit, or cap on to the end of the nerve stump.^11,20^ While these techniques have been effective, nerve grafting and conduits do not protect the nerve end from the external environment, which includes growth factors that promote axon growth. Therefore, over time the axons can grow in an aligned manner all the way through the graft or conduit and then form a neuroma as they exit the open distal end where there is no longer a structure to help facilitate aligned growth.

Capping the nerve end can isolate the axons from external signaling and prevent disorganized growth and neuroma formation. The use of autologous vein and epineurium caps have demonstrated promising results for the prevention of neuroma formation in several studies,^12,21,22^ and without the issues associated with nonbiological materials such as silicone. However, such approaches have their own disadvantages. The harvest of autologous tissue requires extra operative time, introduces risks with donor site morbidity, and can pose an additional technical challenge to the inexperienced surgeon. Moreover, the use of epineurial and vein caps does not guarantee containment of axonal sprouting within the confines of the cap. This is demonstrated by studies showing axon fibers escaping through suture holes or the small, imperceptible openings at the end of a cap, of which the integrity is essentially subject to the technical expertise of the surgeon and dynamics of the local tissue environment.^23^

Therefore, new treatment methods are still needed to prevent neuromas to reduce dependence on postoperative opioid pain management and to improve outcomes for patients with peripheral nerve damage. Consequently, we set out to evaluate chambered capping devices made from porcine small intestine submucosa (pSIS). We hypothesized that the internal chambering would help align and exhaust axonal outgrowth and that pSIS, which has been shown to remodel into a new soft tissue layer,^24^ would help provide a permanent physical barrier that would reduce neurotrophic signaling and protect the nerve end from mechanical stimulation. Taken together, we hypothesized that these material benefits would reduce or prevent neuroma formation and associated pain.

To assess the effects of a medical device for the prevention of neuroma pain, it is important to use a validated and easily reproducible animal model for neuropathic pain. We selected the tibial neuroma transposition (TNT) model, developed by the Belzberg laboratory, for evaluation of allodynia and hyperalgesia^25^ after intervention with our capping device, as this model allows for direct stimulation of the neuroma by way of mechanical or punctate stimulus with a subsequent visible response from the animal. In addition, transection of the tibial nerve in a rat model is supported as a standardized model to assess neuropathic pain within days of surgical intervention.^25,26^ Furthermore, the TNT model ensures that the behavioral responses due to neuroma stimulation are due to hyperalgesia in the nerve rather than incisional pain since the nerve is transposed from its *in situ* position.^25^ Therefore, this model allowed us to include both behavioral and histological endpoints to understand the correlation between the two.

## Materials and Methods

### Preparation of nerve caps

**FIG. 1.**
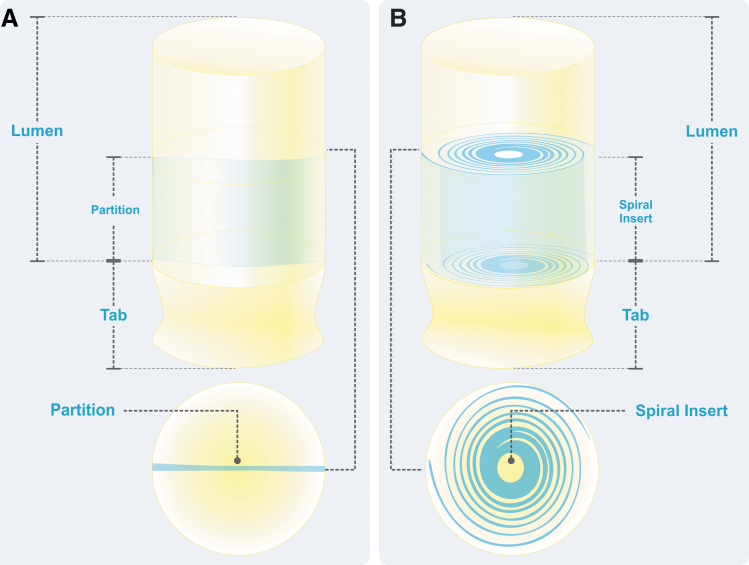
Illustration, nerve cap designs. **(A)** CNC containing a single partition which bifurcates the lumen. **(B)** SNC containing a spiral insert to create multiple chambers within the lumen. CNC, chambered nerve cap; SNC, spiral nerve cap.

pSIS is an extracellular matrix that remodels into a new soft tissue layer.^24^ The pSIS Nerve Caps utilized in this study (Axoguard Nerve Cap^®^; Axogen Corporation, Alachua, FL) were designed to provide channels for organized axonal outgrowth at a terminal nerve stump formed either via a single partition or via a spiral insert (Fig. 1). All caps were 10 mm in length and the internal microarchitecture encompassed the distal 5 mm of the caps. pSIS was utilized to provide a new permanent protective layer of soft tissue while preventing adhesions to the surrounding soft tissue bed.^24^

### Experimental design

Forty-eight male Sprague Dawley rats weighing 257–355 g (62–79 days of age) underwent a TNT procedure, similar to a previously established procedure by Dorsi *et al.*^25^ All animals were deemed healthy and fit for surgery by veterinary staff before the surgical procedure. The nerves were either treated with the chambered nerve cap or spiral nerve cap (CNC group or SNC group, respectively) or they were transposed and secured into the subcutaneous plane without a cap (surgical control [SC] group) (Table 1). An open tube (OT) group was included as a histological control to ensure the pSIS material utilized in the nerve cap remodeled in the same manner as a commercially available pSIS product (Axoguard Nerve Connector; Axogen Corporation).

**Table 1.** Study Groups for Tibial Neuroma Transposition Model

Test group	Initial sample size	Final sample size (initial,* n* = 8 per time point)	
		56 Day (8 weeks)	84 Day (12 weeks)
Chambered nerve cap (CNC)	16	4^a^	7^a^
Spiral nerve cap (SNC)	16	7^b^	7^b^
Surgical control (SC)	16	8	7^c^

^a^ Five animals were euthanized early due to device erosion through the skin.

^b^ Two animals did not have useable histology.

^c^ One animal was inadvertently euthanized early.

Animals were monitored and remained in adequate health for the duration of the study. The rats were sacrificed at either 8 or 12 weeks postoperatively and weights at sacrifice ranged from 377 to 720 g. The experimental protocol adhered to the guidelines of the Animal Welfare Act (CFR 9) and was approved before testing by the Institution Animal Care and Use Committee.

### Surgical procedure

**FIG. 2.**
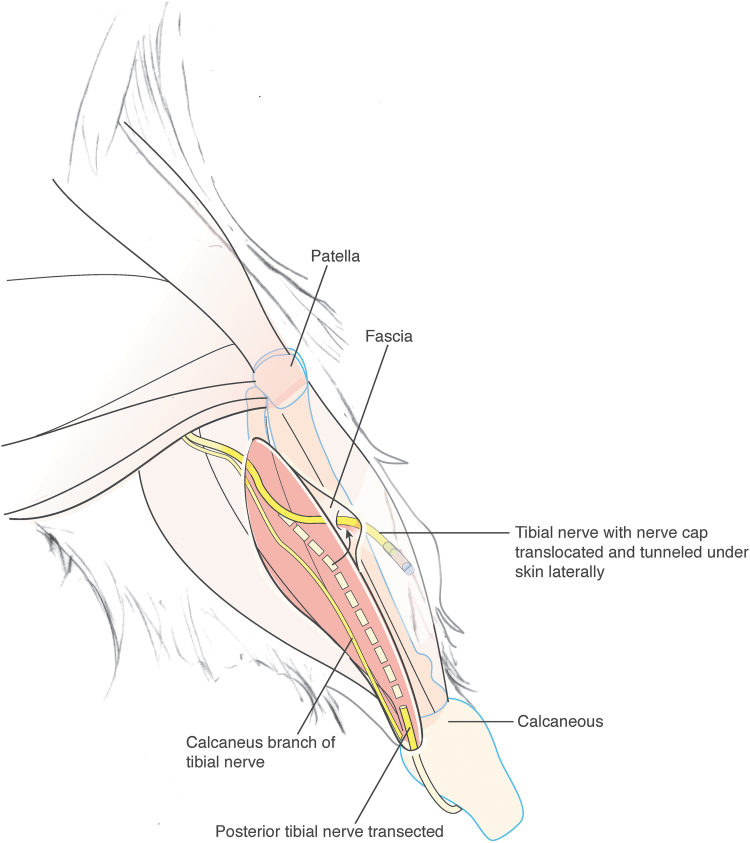
Illustration, medial aspect of rat leg displaying tibial nerve transposition for tibial neuroma transposition model (modified from Dorsi *et al.*^25^).

On the day of the procedure, animals were sedated, anesthetized, and prepared for surgery. Aseptically, the tibial nerve of the left hindlimb of each rat was dissected, ligated, transected and transposed to the lateral hindlimb ∼8 mm superior to the lateral malleolus (Fig. 2). For the SC group, no additional treatments to the tibial nerve were performed following transpositioning^25^ and the distal stump was sutured flush within the subcutaneous space of the lateral hindlimb using appropriate suture material. For both experimental groups and the histological control group (CNC, SNC, and OT respectively), nerve ends were entubulated ∼3 mm into the open lumen of the appropriate cap (chambered or spiral) or OT and secured with nonabsorbable suture. The cap or tube was then secured within a subcutaneous pocket on the lateral hindlimb. The transposition site was indicated by a suture marker and/or a permanent marker dot.

### Behavioral testing

Evaluation of neuropathic pain was conducted through a series of punctate stimuli applied to the treated and untreated (contralateral) legs of each rat. On a weekly basis, a series of 10 trials were performed on each leg for each testing session, as previously outlined by Dorsi *et al.*^25^ The response to each trial was scored with a value of 0–2. Where 0 was no response, 1 was a slow withdrawal of the animal's paw, and 2 was a brisk withdrawal, shaking, licking, or vocalization of the animal. The behavioral testing scores for each animal were represented as the sum of neuroma tenderness values for all 10 trials.

### Histology

The tibial nerve, nerve caps, and OTs were collected, fixed in 10% neutral buffered formalin, embedded in paraffin, and sectioned for histological mounting and staining (Scientific Solutions, Fridley, MN). Paraffin tissue blocks were sectioned such that the explanted nerve stumps and samples were cut along the longitudinal axis of the tissue. Histological sections were taken at three levels through the samples, with levels taken ∼200 μm apart starting from the surface of the sample. At each level, three serial sections were collected and mounted on slides for staining. Slides were configured to ensure that there were three equivalent sets of sections from each sample, which represented each level through the tissue. Slides were stained with hematoxylin and eosin, Masson's Trichrome, or Neurofilament 200 (NF 200).

To determine implant resorption and tissue ingrowth, all slides from the 56-day cohort were compared to the 84-day cohort and were given a score of 0–4, where a score of 0 was considered not present and a score of 4 was considered marked. All scorings were performed by a board-certified veterinary pathologist who was blinded to the surgical groups.

To evaluate the overall tissue response, the veterinary pathologist scored all slides for inflammation, fibrocytes, neovascularization, fatty infiltrates, necrosis, mineralization, hemorrhage, and fibrin deposition using a scale of 0–15. Overall tissue response was expressed as the interpretation of the score, where a score of 0–2.9 was considered a nonirritant, a score of 3.0–8.9 was considered a slight irritant, a score of 9.0–15.0 was considered a moderate irritant, and a score >15.0 was considered a severe irritant. The group averages were then used to calculate the differences between the test groups (CNC and SNC) and the control groups (OT and SC).

In addition, NF 200 stained slides from the SC, CNC, and SNC groups were analyzed for axonal swirling and optical density. Axonal swirling was scored by the veterinary pathologist using a semiquantitative scale ranging from 0 to 4, where a score of 0 was no swirling present and a score of 4 was marked swirling. Optical density of NF 200 stained axons was determined using three randomly selected areas in the distal portion of the nerve stump, which were analyzed for axonal density using Image-Pro Premier 9.2 (Media Cybernetics, Rockville, MD).

### Statistical analysis

General summary statistics for numeric data of inflammation, fibrocytes, neovascularization, fatty infiltrates, necrosis, mineralization, hemorrhage, fibrin deposition, tissue ingrowth, and implant resorption included the mean and the standard deviation. All statistical evaluations were tested at a 0.05 significance level. Axonal swirling statistical evaluations were analyzed using SAS v. 9.4 (SAS Institute, Inc., Cary, NC).

Behavioral testing scores of left and right hindlimbs were analyzed for all animals enrolled in the study to determine whether the mean scores were the same across all groups. Comparisons of the mean scores for the individual hindlimb sides, left or right, between the treatment and control groups was conducted with a Kruskal-Wallis test (a nonparametric version of analysis of variance). A Kruskal-Wallis test was also used to perform a three-group comparison at all timepoints. A *post hoc* Tukey and Kramer-Nemenyi multiple comparison tests were used to compare differences between the groups at all timepoints with a significant group difference on the Kruskal-Wallis test.

Optical density data were also analyzed using a Kruskal-Wallis test as well as *post hoc* multiple comparison tests using Tukey and Kramer-Nemenyi tests. All data are presented as the mean ± standard error of the mean. Pearson correlations were run to determine any correlation between axonal swirling and optical density and behavior and optical density.

## Results

### Tissue response

The overall tissue response showed no statistical differences across all groups at 8 weeks and all implants were noted as being nonirritants. Overall average tissue responses at 8 weeks were 2.7 for the CNC group, 1.7 for the SNC group, and 1.3 for the OT group. After 12 weeks of recovery, the overall tissue response showed no statistical differences across all groups and all implants were again noted as being nonirritants. Overall average tissue responses at 12 weeks were 0.0 for the CNC group, 1.4 for the SNC group, and 2.1 for the OT group.

All samples showed minimal to mild implant resorption by 12 weeks, with notable amounts of the implant material remaining. The average 12-week implant resorption score was 1.1 for the CNC group, 1.5 for the SNC group, and 1.3 for the OT group. The tissue ingrowth scores of these samples also showed no statistical differences across all groups by the 12-week time point, with average scores of 1.1 for the CNC group, 1.6 for the SNC group, and 1.5 for the OT group. These results indicate that pSIS, when used in the two different nerve cap configurations, remodels in the same manner as the commercially available pSIS tube.

### Axonal swirling

**FIG. 3.**
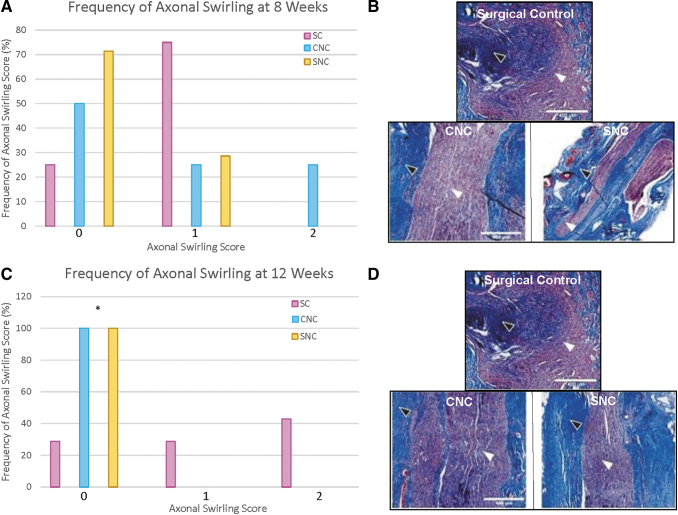
Axonal swirling analysis **(A)** at 8 weeks, there was no statistically significant difference among the groups, with more than 75% of all scores between 0 and 1. **(B)** Representative micrographs of 8-week Masson's trichrome staining. Axonal swirling can be observed by axonal growth patterns (*white arrowheads*) through the collagen matrix (*black arrowheads*). **(C)** At 12 weeks, axonal swirling was scored a 0 in all animals in both nerve cap groups. The SC group showed significantly higher axonal swirling compared to both the CNC and SNC groups (*p = 0.0210 and 0.0210, respectively). **(D)** Representative micrographs of 12-week Masson's trichrome staining. SC, surgical control.

Axonal swirling scores at 8 weeks also showed no statistical differences across the three groups. However, trends of higher axonal swirling scores were more frequently noted in the SC group (75%), compared to the CNC group (50%) and the SNC group (28.6%) (Fig. 3A). At 12 weeks, there was a significant difference in axonal swirling score frequency with the SC group (71.5%) having significantly higher axonal swirling scores more frequently than the CNC and SNC groups (Fisher's exact test *p* = 0.02). The CNC and SNC groups showed no identifiable axonal swirling in 100% of the animals (Fig. 3C).

### Optical density

**FIG. 4.**
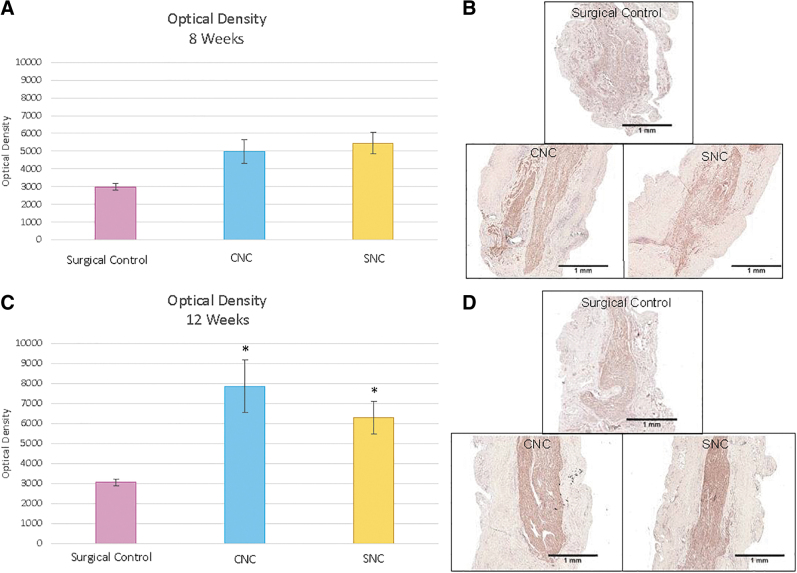
Optical density values (mean ± SEM) **(A)** optical density values within the distal nerve segment at 8 weeks. There were no significant differences between the three groups. **(B)** Representative micrographs of 8-week neurofilament staining that were used for optical density measurements. **(C)** Optical density values within the distal nerve at 12 weeks. The CNC group had a statistically higher optical density than the SC group (*p = 0.0004). The difference between the SNC and SC group was also statistically significant (*p = 0.0002). There was no difference between the CNC and SNC groups (*p* = 0.9787). **(D)** Representative micrographs of 12-week neurofilament staining that were used for optical density measurements. SEM, standard error of the mean.

To obtain a histological correlate of neuroma formation, optical density measurements of the regenerating nerve stumps were performed.^11^ There is an inverse relationship between axon density and collagen, therefore, a decrease in axonal optical density measurements is indicative of increased collagen deposition and associated neuroma formation. At 8 weeks, there were no significant differences between the groups. The average optical densities were 2984 ± 191 for the SC group, 4961 ± 667 for the CNC group, and 5439 ± 603 for the SNC group (Fig. 4).

At 12 weeks, the SC group showed significantly lower axonal optical density of neurites extending within regenerating nerve stumps compared to the CNC and SNC groups (*p* < 0.001, Tukey and Kramer-Nemenyi test, Fig. 4C). At 12 weeks, the average axonal optical density of the SC was 3062 ± 166 compared to 7858 ± 1322 for the CNC group and 6289 ± 824 for the SNC group. There was no significant difference in the optical density between the CNC and SNC groups (Fig. 4C, *p* = 0.9787). This indicates that both the CNC and SNC groups had an increased density of axonal outgrowth and less collagenous tissue present in the regenerating nerve stumps compared to the SC group. These results, taken together with the axonal swirling results, indicate that the Nerve Caps were able to reduce or eliminate neuroma formation.

### Behavioral pain response

**FIG. 5.**
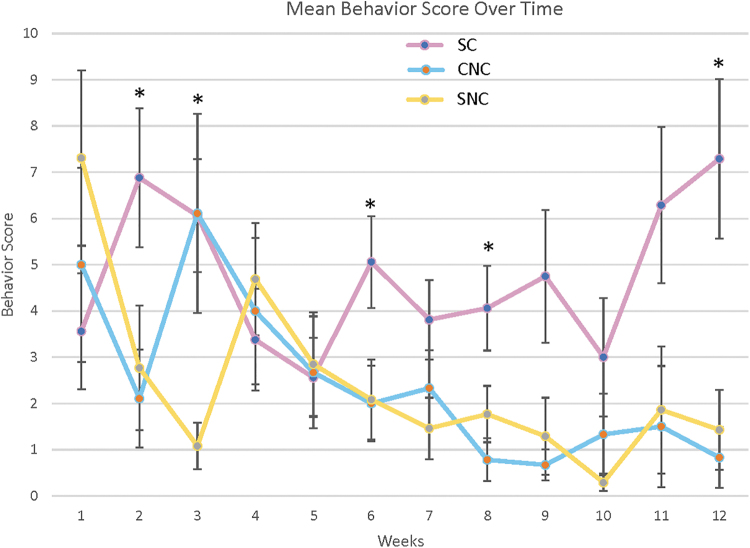
Behavioral pain responses over time, recorded after punctate stimulus of tibial nerve stumps (mean ± SEM). Significant differences were seen between the groups at 2, 3, 6, 8, and 12 weeks (three-groups Kruskal-Wallis, *p-value <0.05). In addition, at 2 and 8 weeks, the SC group had a significantly higher pain response than the CNC group, at 3 weeks the SC group had a significantly higher pain response than the SNC group and at 12 weeks the SC group had a significantly higher pain response than both the CNC and SNC groups (Tukey and Kramer-Nemenyi, *p-value <0.05).

For the first 6 weeks after surgery, the pain response scores of all three groups were highly variable (Fig. 5). Pain during this time was most likely due to postsurgical pain. At 2 weeks, there was a significant difference between the groups using a three-group Kruskal-Wallis test (*p* = 0.02). In addition, there was a significant difference between the SC and the CNC groups with the SC group exhibiting a higher pain response using a multiple comparisons test (*p* = 0.035, Tukey and Kramer-Nemenyi Test).

At 3 weeks, there was a significant difference between the groups using a three-group Kruskal-Wallis test (*p* = 0.009) and a significant difference between the SC and SNC groups using a multiple comparisons test (*p* ≤ 0.05). At 6 weeks, there was a significant difference between the three groups using a three-group Kruskal-Wallis test (*p* = 0.04), but no significant difference was seen using a multiple comparisons test (Tukey and Kramer-Nemenyi). By 8 weeks, there was a significant difference between the three groups using the three-group Kruskal-Wallis test (*p* = 0.04) as well as a significant difference between the SC and CNC group using a multiple comparisons test (*p* = 0.048, Tukey and Kramer-Nemenyi test).

By 12 weeks, there was a significant difference between the groups using the three-group comparison Kruskal-Wallis test (*p* = 0.008) as well as using the multiple comparisons test with the SC group exhibiting a significantly higher pain response than both the CNC and SNC groups (*p* = 0.021 and 0.03, respectively). No significant differences between the CNC and SNC groups were seen at any timepoint. The variability in pain response scores before the 12-week timepoint was expected since neuromas typically take at least 12 weeks to form.^27^ This behavioral pain response data indicate the Nerve Cap's potential to reduce or eliminate symptomatic neuroma formation—for both the partition and spiral insert designs.

## Discussion

Since the popularization of collagen conduits for nerve repair in the mid-1990s,^28,29^ the use of biomaterials in nerve repair has steadily increased in popularity with the options expanding beyond collagen to include other materials such as polyglycolic acid,^30^ pSIS,^31^ and processed nerve allografts.^32^

Originally utilized in end-to-end nerve reconstruction after trauma, these biomaterials have recently been recognized as having a role in the surgical management of nerve pain and painful neuroma.^33^ For example, nerve allograft has been used to reconstruct nerves after resection of painful neuromas-in-continuity^33^ and conduits, when placed over a nerve coaptation, have been shown to reduce the likelihood of pain at the coaptation by 50%.^34^ However, although nerve caps have been utilized as a method to try and prevent neuroma development in the past, they were either made from nonbiologic materials such as silicone or from autologous tissues such as vein or epineurium.^12,21^ These treatments have not gained in popularity, likely due to the fact that silicone can cause problematic reactions when implanted in the body and autologous tissue requires extra operative time, introduces risks with donor site morbidity, and can pose an additional technical challenge while not guaranteeing the containment of axons within the cap.^23^ Therefore, we chose to utilize pSIS, a known biomaterial that is well tolerated and has been previously utilized around nerve,^24,31,35^ in the design of our cap.

pSIS serves as a biologic scaffold that revascularizes and remodels into a new, permanent soft tissue layer^24^ unlike collagen, which simply degrades.^36^ This new soft tissue layer that forms can help to reduce neurotrophic signaling from the surrounding environment while also providing permanent protection of the nerve end from mechanical stimulation. This is consistent with the behavioral data obtained in this study, which demonstrated that the rats in the CNC and SNC groups had significantly lower behavioral pain scores in response to a mechanical stimulus indicating that they were less sensitive (i.e., exhibited less pain) to mechanical stimulation of the terminal nerve stump over the course of the study compared to the SC group. pSIS also protects the nerve from soft tissue attachments therefore minimizing the potential for pain due to scar formation causing pressure and traction on the neuroma.^24^

In addition to choosing a biomaterial that will remodel into a new soft tissue layer, we included an internal microarchitecture in the design. The CNCs contained a pSIS partition that ran down the center of the cap, dividing the cap into two separate chambers. The SNCs contained a pSIS spiral insert running from the inner circumference at the end of the cap, toward the center. Internal microarchitecture within the nerve caps served two different purposes. First, internal microstructure has been shown to better support axonal growth and limit axonal dispersion by helping to align axonal outgrowth by providing a microenvironment with which the axons can interact,^37^ which is supported by the results of this study. This study demonstrated that at 12 weeks both the CNC and SNC groups showed an increase in axonal optical density compared to the SC group, which had a notable decrease in the axonal optical density measurements at the site of regenerating nerve stumps. This decrease in axonal optical density is associated with increased collagen deposition and disorganized axonal outgrowth, features that signify neuroma formation.^11^

In addition, the partition and spiral inserts served as a method to prevent the terminated nerve end from being placed too far into the cap. This gave the nerves a predefined “runway” length to allow for aligned axonal outgrowth and eventual axonal exhaustion.^38,39^ The “runway” length of 5 mm was based on several studies that showed very minimal axon infiltration past 5 mm in hollow tubes used to repair nerve defects.^39,40^ Five millimeter was considered a “worst-case” scenario and nerve caps for clinical use, which are available in diameters up to 4 mm, have been designed with a 10 mm runway to ensure axonal exhaustion.

One limitation of this study was the use of the TNT model itself. The nature of the model and the transposition of the nerve, while having the benefit of allowing for mechanical stimulation testing, only allows for comparison to simple neurectomy and not to other methods such as burying in muscle or bone. In addition, rodents have a very thin skin envelope compared to humans and this model necessitates that the device be implanted directly under the skin in a site near a joint with very limited space, which likely accounts for the device erosions seen in this study. This is different from the intended clinical use where the cap would have sufficient healthy soft tissue coverage. Furthermore, the model requires a suture through the skin to anchor and mark the location of the nerve end. This may have caused some irritation and chewing of the suture material, which may have also contributed to the erosions.

Use of the 12-week timepoint could be seen as another limitation of this study. However, Oliveira *et al.*, studied the time course of neuroma development in rodents and demonstrated that neuroma formation begins as early as 4 weeks postamputation with fully formed neuromas present by 12 weeks.^27^ This is consistent with our findings of histological correlates of neuroma formation in the SC group at 12 weeks. Finally, we saw no correlation between axon swirling and optical density scores or between optical density and behavior scores. However, the lack of correlation is likely due to the study being underpowered for these types of statistics. This study was powered for equivalence and correlations typically require a larger sample size than equivalence.

## Conclusion

Histologically, neuromas are characterized by a bulbous mass of axons entangled in connective tissue. The functional result of these entrapped axons is pain and dysesthesia.^15^ This study demonstrated that isolating terminal nerve stumps within nerve caps containing an internal structure resulted in reduced pain sensitivity to mechanical stimuli exhibited by the animals, less axonal swirling, and higher axonal optical density (axon to collagen ratio) in the regenerating nerve stumps compared to the SC group. Taken together, these results indicate that entubulation of terminal nerve stumps within a chambered or spiralized nerve cap reduces the risk of developing a painful neuroma.^11,41^ Future application of the nerve cap in the treatment of residual limb patients may decrease the incidence of painful neuromas, which has the potential to improve an amputee's tolerance of his or her prosthetic and subsequent rehabilitation outcomes.
